# MicroRNA Markers for the Diagnosis of Pancreatic and Biliary-Tract Cancers

**DOI:** 10.1371/journal.pone.0118220

**Published:** 2015-02-23

**Authors:** Motohiro Kojima, Hiroko Sudo, Junpei Kawauchi, Satoko Takizawa, Satoshi Kondou, Hitoshi Nobumasa, Atsushi Ochiai

**Affiliations:** 1 Department of Pathology, National Cancer Center Hospital East, Kashiwa, Chiba, Japan; 2 New Frontiers Research Laboratories, Toray Industries, Inc., Kamakura, Kanagawa, Japan; 3 New Projects Development Division, Toray Industries, Inc., Kamakura, Kanagawa, Japan; Baylor University Medical Center, UNITED STATES

## Abstract

It is difficult to detect pancreatic cancer or biliary-tract cancer at an early stage using current diagnostic technology. Utilizing microRNA (miRNA) markers that are stably present in peripheral blood, we aimed to identify pancreatic and biliary-tract cancers in patients. With “3D-Gene”, a highly sensitive microarray, we examined comprehensive miRNA expression profiles in 571 serum samples obtained from healthy patients, patients with pancreatic, biliary-tract, or other digestive cancers, and patients with non-malignant abnormalities in the pancreas or biliary tract. The samples were randomly divided into training and test cohorts, and candidate miRNA markers were independently evaluated. We found 81 miRNAs for pancreatic cancer and 66 miRNAs for biliary-tract cancer that showed statistically different expression compared with healthy controls. Among those markers, 55 miRNAs were common in both the pancreatic and biliary-tract cancer samples. The previously reported miR-125a-3p was one of the common markers; however, it was also expressed in other types of digestive-tract cancers, suggesting that it is not specific to cancer types. In order to discriminate the pancreato-biliary cancers from all other clinical conditions including the healthy controls, non-malignant abnormalities, and other types of cancers, we developed a diagnostic index using expression profiles of the 10 most significant miRNAs. A combination of eight miRNAs (miR-6075, miR-4294, miR-6880-5p, miR-6799-5p, miR-125a-3p, miR-4530, miR-6836-3p, and miR-4476) achieved a sensitivity, specificity, accuracy and AUC of 80.3%, 97.6%, 91.6% and 0.953, respectively. In contrast, CA19-9 and CEA gave sensitivities of 65.6% and 40.0%, specificities of 92.9% and 88.6%, and accuracies of 82.1% and 71.8%, respectively, in the same test cohort. This diagnostic index identified 18/21 operable pancreatic cancers and 38/48 operable biliary-tract cancers in the entire cohort. Our results suggest that the assessment of these miRNA markers is clinically valuable to identify patients with pancreato-biliary cancers who could benefit from surgical intervention.

## Introduction

Pancreatic cancer is one of the most lethal cancers. Most pancreatic cancers do not accompany any particular clinical symptoms in the early stage, permitting the cancers to progress undetected. In addition, ambiguous radiological images of cancerous lesions and inflammatory conditions in the pancreas prevent pancreatic cancers from being correctly identified. Furthermore, the anatomical location of the pancreas, deep in a retroperitoneal space surrounded by many other organs, hinders the acquisition of a biopsy. All of these factors prevent the early detection of pancreatic cancer. The American Cancer Association estimated that 40,000 people would die of pancreatic cancer in 2014 in the United States [1]. The five-year survival rate for patients with exocrine pancreatic cancer is estimated at 14% for stage IA, but it drops to 1% for stage IV [1]. Because the most promising treatment for pancreatic cancer is surgical resection, detecting pancreatic cancer at surgically resectable stages is crucial for improving the survival rate of patients with pancreatic cancer. From this point of view, the screening of the early stages of pancreatic or biliary-tract cancers is imperative.

As a diagnostic screening method for pancreatic cancer, ultrasound is one of the most prevalent tests performed. However, this image analysis has its difficulty in differentiating non-malignant tissue from malignant tissue [2]. In addition, many tumor-associated antigens have been studied in connection with pancreatic cancer. The most validated and clinically useful biomarker is carbohydrate antigen (CA) 19–9; however, CA19-9 is known to be up-regulated in other inflammatory conditions, and its low positive predictive value makes it a poor biomarker for screening, limiting its current use mostly to the post-surgical monitoring of progressed pancreatic cancers [3, 4]. Today, there is no effective method to detect early, surgically resectable pancreatic cancers with sufficient diagnostic accuracy.

Recently, microRNAs (miRNAs) have been reported as potential biomarkers for various types of cancers. Using plasma samples from 50 cancer patients and ten healthy control subjects, as well as chemo-resistant pancreatic cell lines, Ali *et al*. suggested that serum miR-21 and other miRNAs could predict the aggressiveness of pancreatic cancer [5]. Ganepola *et al*. examined a dozen of plasma samples each from patients with and without pancreatic cancer, and concluded that three miRNAs, miR-642b, miR-885-5p, and miR-22, were more than 90% sensitive and specific in the diagnosis of pancreatic cancer [6]. Similarly, Li’s group examined 20 or less serum samples each from patients with pancreatic cancer and control, respectively, as the biomarker discovery cohort, and found that multiple miRNAs including miR-1290 were useful in the early detection of pancreatic cancer [7]. More recently, researchers have analyzed more than 100 blood samples each from pancreatic cancer patients and controls, and some found miR-155, miR-181a, miR-181b and miR-196a [8], and others found miR-20a, miR-21, miR-24, miR-25, miR-99a, miR-185, and miR-191 [9] for blood miRNA markers. It should be noted that each of the previous studies suggested different circulating miRNA markers for pancreatic cancer. The discrepancies of previous studies could be attributed to various empirical factors that include the blood sample types (PBMC, plasma or serum), different detection technologies (PCR, microarray or sequencer), and heterogeneity of the sample cohorts. Especially, the sufficient size and the diversity of the sample cohorts are critical in biomarker research not only for the targeted group but also for the control group.

Here, we examined the expression profiles of comprehensive serum miRNAs from the largest cohorts of patients ever attempted: 100 patients with pancreatic cancer, 98 patients with biliary-tract cancer, 150 healthy control patients, 21 patients with non-malignant abnormalities in the pancreas or biliary tract, and 202 patients with other types of cancers. A highly sensitive microarray permitted the simultaneous analysis of more than 2,500 miRNAs that were recently updated in the miRBase (release 20), and serum samples from patients with the various clinical conditions allowed us to evaluate a wide range of diagnostic specificity in the detection of pancreato-biliary cancer.

By combining eight miRNA markers, we were able to detect patients with pancreato-biliary cancers among those who were healthy, had non-malignant abnormalities or had other types of cancers, with a diagnostic accuracy of 91.6% and AUC of 0.953.

## Methods

### Ethics statement

This research on human subjects was approved by the National Cancer Center Hospital East Institutional Review Board (2010-096) and by the Human Tissue Samples Ethics Committee for R&D, Toray Industries Inc. (HC2013-4, 128 and HC2014-4). Written informed consent was obtained from each participant.

### Clinical samples

Blood samples were obtained from a total of 421 patients who were admitted to the Japanese National Cancer Center Hospital East during the years 2010 to 2012. One hundred patients with pancreatic cancer, 98 patients with biliary-tract cancer, 50 patients with colon cancer, 50 patients with stomach cancer, 50 patients with esophageal cancer, 52 patients with liver cancer, and 21 patients with non-malignant pancreatic or biliary-tract diseases were registered in the Biobank and selected for use in this study. For pancreatic and biliary-tract cancers, patients with the following characteristics were excluded: (i) patients with intraductal papillary mucinous neoplasm, (ii) patients simultaneously or previously diagnosed with advanced cancer in another organ, (iii) patients with cancer uncertain to be either pancreatic or biliary, and (iv) patients with special histology other than adenocarcinoma. All the patients were histologically confirmed, and those with cancer were pathologically diagnosed as having adenocarcinoma. All blood samples from cancer patients were taken before any treatment except one pancreatic cancer case with ypStage I which had received therapeutic intervention. The detailed information about these patients is shown in Table 1B-D.

Control blood samples were obtained from healthy individuals recruited from the Japanese affiliate companies of Toray Industries Inc. in 2013. Inclusion criteria for healthy control individuals were age greater than 60 years, no history of any cancer, and no hospitalization during the last 3 months (Table 1A).

**Table 1:** Demographics of healthy control individuals (A), patients with pancreatic cancer (B), patients with biliary-tract cancer (C), patients with non-malignant abnormalities (D), and (E) patients with other types of cancer.

A) Healthy control individuals		
Number of patients	-	150
Gender	Male	136
	Female	14
Age	median (range)	62 (60–69)
Serum CA19-9	median (range) U/mL	7.5 (0.5–98)
Serum CEA	median (range) ng/mL	1.9 (0.4–7.2)
B) Patients with pancreatic cancer		
Number of patients	-	100
Gender	Male	64
	Female	36
Age	median (range)	68 (33–81)
Tumor stage	pStage I	1
	pStage II	18
	cStage III	27
	cStage IV	54
Pathological location of tumor	Pancreatic head	46
	Pancreatic body	32
	Pancreatic tail	20
	Pancreatic body/tail	2
Serum CA19-9	median (range) U/mL	580 (0.1–971,000)
Serum CEA	median (range) ng/mL	5.5 (0.7–41.7)
Serum D-Bilirubin	median (range) ng/dL	0.2 (0–16.3)
C) Patients with biliary-tract cancer		
Number of patients	-	98
Gender	Male	63
	Female	35
Age	median (range)	67 (33–86)
Operability	Operable	48
	Inoperable	50
Pathological location of tumor	Intrahepatic bile duct	30
	Extrahepatic bile duct	22
	Gall bladder	29
	Hilar bile duct	9
	Ampulla of Vater	8
Serum CA19-9	median (range) U/mL	70.9 (0.1–68,120)
Serum CEA	median (range) ng/mL	3.2 (0.2–2,030)
Serum D-Bilirubin	median (range) ng/dL	0.2 (0.1–17.2)
D) Patients with non-malignant abnormalities in the pancreas or biliary tract		
Number of patients	-	21
Gender	Male	13
	Female	8
Age	median (range)	56 (33–76)
Clinical condition	Chronic pancreatitis	9
	Chronic cholecystitis	5
	Abnormal conjunction of pancreatic duct and bile duct	2
	Intrahepatic stone	1
	High level of serum Dupan-2	1
	Bile-duct enlargement	1
	Abnormal pancreatic histology	1
	Acute weight loss	1
Serum CA19-9	median (range) U/mL	10 (0.1–255)
Serum CEA	median (range) ng/mL	1.9 (0.7–12.7)
E) Patients with other types of cancers		
E) -1 Colon cancer		
Number of patients	-	50
Gender	Male	33
	Female	17
Age	median (range)	63 (32–82)
Tumor stages	0	0
	I	18
	II	11
	III	16
	IV	0
	Undetermined	5
Serum CA19-9	median (range) U/mL	13.2* (0.1–3524)
Serum CEA	median (range) ng/mL	2.8 (0.1–268)
E)-2 Stomach cancer		
Number of patients	-	50
Gender	Male	31
	Female	19
Age	median (range)	69 (35–85)
Tumor stages	0	0
	I	35
	II	7
	III	8
	IV	0
	Undetermined	0
Serum CA19-9	median (range) U/mL	10.3 (0.1–77.4)
Serum CEA	median (range) ng/mL	2.7 (0.3–17.5)
E)-3 Liver cancer		
Number of patients	-	52
Gender	Male	40
	Female	10
Age	median (range)	69 (36–84)
Tumor stages	0	0
	I	24
	II	14
	III	10
	IV	2
	Undetermined	0
Serum CA19-9	median (range) U/mL	13.1* (0.1–105)
Serum CEA	median (range) ng/mL	3.2* (0.6–12.1)
E)-4 Esophageal cancer		
Number of patients	-	50
Gender	Male	44
	Female	6
Age	median (range)	69 (47–87)
Tumor stages	0	0
	I	1
	II	5
	III	16
	IV	27
	Undetermined	1
Serum CA19-9	median (range) U/mL	-*
Serum CEA	median (range) ng/mL	3.1* (0.5–14.2)

*CA19-9 and/or CEA scores were not available in some cases.

All peripheral blood samples were processed to serum within a day of acquisition, and serum biomarkers including CA19-9 and CEA were measured before the remaining serum samples were stored at -80°C for miRNA analysis.

Formalin Fixed Paraffin Embedded (FFPE) samples of pancreatic tumors and adjacent normal tissues were obtained from 10 randomly chosen patients with pancreatic cancer (one case of stage IIA, eight cases of stage IIB, and one case of stage III). In the FFPE sections with the size of 10×10μm, tumor area was marked by ink using the neighboring slides that were H.E. stained. The tumor area and the adjacent normal tissue were then macroscopically dissected.

### miRNA expression analysis by microarray

For each clinical group, the miRNA expression analysis was performed simultaneously regardless of the training cohort or the test cohort. Total RNA was extracted from each 300-μL serum sample using “3D-Gene” RNA extraction reagent from a liquid sample kit (Toray Industries, Inc., Tokyo, Japan). Total RNA was also obtained from the FFPE sections using the “Arcturus” “Paradise” Extraction and Isolation kit (Life Technologies, Carlsbad, CA, U.S.A.) in accordance with the manufacturer's instructions. Comprehensive miRNA expression analysis was performed using a “3D-Gene” miRNA Labeling kit and a “3D-Gene” Human miRNA Oligo Chip (Toray Industries, Inc.), which was designed to detect 2,555 miRNAs registered in the miRBase release 20 (http://www.mirbase.org/).

Individual miRNAs were regarded as present if the corresponding microarray signals were more than (the mean + 2x standard deviation) of the negative control signals of which the top and bottom 5% ranked by signal intensity were removed. Once the miRNA was regarded as present, the miRNA signal was subtracted with the mean signal of the negative controls of which the top and bottom 5% ranked by signal intensity were removed. When the signal became a negative value (or was undetected) after the background subtraction, the value was replaced by the number that was the lowest signal intensity on the microarray minus 0.1 on a log_2_ scale. In order to normalize the signals across the different microarrays tested, quantile normalization was performed [10]. All microarray data from this study are in agreement with the Minimum Information About a Microarray Experiment (MIAME) and are publicly available through the Gene Expression Omnibus (GEO) database (http://www.ncbi.nlm.nih.gov/projects/geo/) under the accession number GSE59856.

### Statistical analysis

In this study, the miRNA expression profiles of the following five paired-clinical conditions were statistically compared: i) pancreatic cancer versus healthy control, ii) biliary-tract cancer versus healthy control, iii) pancreato-biliary cancer versus non-malignant abnormalities, iv) pancreato-biliary cancer versus other types of cancer, and v) pancreato-biliary cancer versus all other clinical conditions (healthy control, non-malignant abnormalities, and other types of cancer). For each condition, patients with pancreatic and/or biliary-tract cancer were regarded as the cancer group, and the other groups were regarded as the control.

After all microarray experiments were performed, the samples of each clinical condition were randomly divided into a training cohort (2/3 of the samples) and a test cohort (1/3 of the samples) by computation. The clinical characteristics of the training cohort and the test cohort was examined and confirmed that there was no significant difference in these two cohorts. The training cohort was used to select significant miRNA markers and to define their discriminant functions, and the test cohort which is independent from the training cohort was used to validate the diagnostic performance of the selected miRNA markers based on the same statistical significance. The analyses of the training cohort and the test cohort were programmed in advance and sequentially performed without overcrossing those cohorts. In the selection process of miRNA markers, any two clinical groups were compared using two-sided Student’s t-test, and a Bonferroni-corrected p-value of <0.01 was regarded as statistically significant. For FFPE analysis, since only eight miRNAs that were preselected was analyzed, Bonferroni correction was not applied to p-value. In order to obtain robust biomarkers, only miRNAs that showed a signal value of 2^6^ in more than 50% in either clinical group being compared were selected for further analysis. Using these miRNAs, a Fisher’s linear discriminant analysis was performed, and the resulting diagnostic sensitivity, specificity and accuracy were calculated for each miRNA marker or combination of miRNA markers.

When expressions of multiple miRNAs were used in the algorithm development, the discriminant functions were created with Fisher’s linear discriminant analysis. The resulting values of the discriminant functions were called as diagnostic indices. Clinical samples that showed the indexed score over 0 were classified to pancreato-biliary cancer, and samples that showed the indexed score under 0 were classified to non-pancreato-biliary cancer (or other clinical conditions). As another statistical tool to analyze biomarkers’ diagnostic performance, Receiver Operating Characteristic (ROC) analysis and its Area Under the Curve (AUC) values were also used.

All computations were performed using R version 3.0.2 (R Foundation for Statistical Computing, http://www.R-project.org), MASS package version 7.3–30 [11] and bee swarm package version 0.1.6 [12].

## Results

### Clinical characteristics of the healthy control individuals and the patients with pancreatic cancer, biliary-tract cancer, other types of cancer, or non-malignant abnormalities in pancreato-biliary tract

The characteristics of 150 healthy control individuals, 100 pancreatic cancer patients, 98 biliary-tract cancer patients, 21 patients with non-malignant abnormalities in those organs, and 202 patients with other types of cancers are presented in Table 1A-E. The median age of the healthy controls was slightly less (62 years of age) than that of the patients with pancreatic cancer (68 years of age) or with biliary-tract cancer (67 years of age). In addition, the healthy control group was clearly dominated by males (91%), while the patients with cancers were less so (64% male for both the pancreatic and biliary-tract cancer groups).

All 100 pancreatic cancer patients had pancreatic ductal adenocarcinoma. Twenty-one out of 100 patients with pancreatic cancer underwent surgical resection (1 ypStage I, 1 ypStage IIB, 17 pStage II, 1 cStage III and 1 cStage IV). All other patients with pancreatic cancer who did not undergo surgical resection were classified as cStage III or IV. On the other hand, biliary-tract cancer cases were classified simply by operable and unoperable cases, due to the complex TNM staging system and distinct biological identities across different primary sites (intrahepatic bile duct, extrahepatic bile duct, gall-bladder, etc.) within biliary tract.

### Comparison of pancreatic cancer or biliary-tract cancer with healthy control

**Fig 1:**
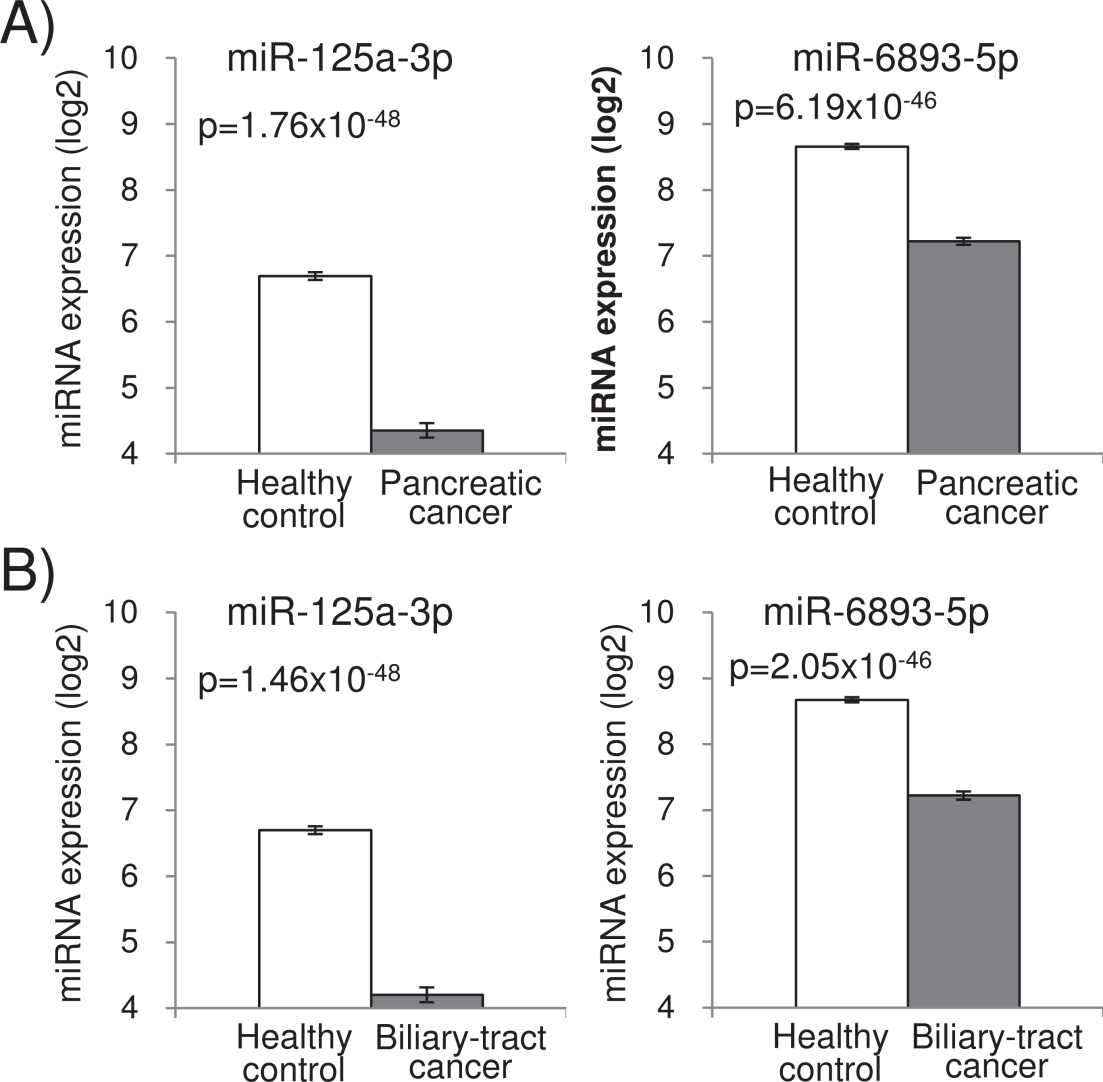
Expression signals of miR-125a-3p and miR6893-5p that showed the smallest p-values in comparison of pancreatic cancer and healthy control (A), or in comparison of biliary-tract cancer and healthy control (B) in the training cohort. The error bars indicate standard error. The p-values were Bonferroni-corrected.

Using the training cohort, the patients with pancreatic cancer were statistically compared with the healthy control individuals, and 120 miRNAs showed a potential to separate those two clinical groups with Bonferroni-corrected p-value of less than 0.01 by Student’s t-test. Out of the 120 miRNAs selected in the training cohort, 81 miRNAs were statistically validated in the test cohort (p<0.01). Among those, 40 miRNAs were up-regulated and 41 miRNAs were down-regulated in pancreatic cancer. The list of these selected miRNAs, their p-values, the expression levels and the diagnostic performance were provided in S1 Table. Fig. 1A shows expression signals of miR-125a-3p and miR-6893-5p which showed the smallest p-values in comparison of pancreatic cancer patients and the healthy control individuals in the training cohort, and were also validated in the test cohort.

The best marker, miR-125a-3p, was able to detect 30 of 33 (90.9%) pancreatic cancers in the test cohort. In contrast, the widely used blood biomarkers for pancreatic cancer, CEA and CA19-9, detected 52 (52.0%) or 77 (77.0%), respectively, out of 100 pancreatic cancers in the entire cohort. Overall, 40 of 81 validated miRNA marker candidates for pancreatic cancer showed sensitivities higher than that of CA19-9 in the test cohort (S1 Table).

Similarly, we performed a comparative analysis of the patients with biliary-tract cancer and the healthy control individuals. One hundred twenty-four miRNAs showed statistical significance (p<0.01) in the training cohort, and out of those 124 miRNAs, 66 miRNAs were validated in the test cohort (S1 Table). Among those, 30 miRNAs were up-regulated and 36 miRNAs were down-regulated in biliary-tract cancer. Expression signals of miR-125a-3p and miR-6893-5p which showed the smallest p-values in comparison of biliary-tract cancer and healthy control in the training cohort and also were validated in the test cohort were shown in Fig. 1B. Again, the best marker to detect biliary-tract cancer was miR-125a-3p, and was able to detect 32 of 33 (97.0%) in the test cohort. In contrast, CEA and CA19-9 detected 30 (30.6%) and 62 (63.3%), respectively, out of 98 biliary-tract cancers in the entire cohort. Overall, 61 of 66 validated miRNA marker candidates for biliary-tract cancer, showed sensitivity higher than that of CA19-9 (57.1%) in the test cohort (S1 Table).

We also found that, despite apparent histological differences, a number of miRNAs were selected as markers not only for pancreatic cancer but also for biliary-tract cancer; of the 81 miRNAs selected as marker candidates for pancreatic cancer, 55 (67.9%) were also selected as marker candidates for biliary-tract cancer. In contrast, when miRNAs that could discriminate pancreatic cancer from biliary-tract cancer were sought, none reached statistical significance. The miRNA that showed the smallest Bonferroni-corrected p-value (0.071) in comparison of pancreatic and biliary-tract cancer was miR-1227-5p; however, that miRNA was selected as a marker both for pancreatic cancer (ranked 78) and for biliary-tract cancer (ranked 46) (S1 Table). Those results led us to conclude that the differentiation of pancreatic cancer and biliary-tract cancer by serum miRNAs would be difficult.

This strong overlap in miRNA expression profiles between pancreatic cancer and biliary-tract cancer led us suspect that these miRNA markers were affected by jaundice, a common clinical condition over pancreas and biliary-tract. Therefore, we examined a jaundice marker, blood D-bilirubin, in pancreato-biliary cancer patients. Eighty-one of 100 pancreatic cancer patients and 78 of 98 biliary-tract cancer patients had blood D-bilirubin under the cutoff value of 0.8 mg/dL, and the median value of blood D-bilirubin concentration was 0.2 mg/dL both for pancreatic and biliary tract cancer patients, suggesting that a majority of these patients were non-jaundice. Furthermore, none of those 55 miRNA markers that overlapped in pancreatic cancer and biliary-tract cancer was correlated with D-bilirubin level (the highest Pearson’s correlation was -0.37). These results confirmed that the common miRNAs selected above were not likely a marker for jaundice but a marker for pancreato-biliary cancer.

Based on these observations, we therefore decided to pursue the markers that would simultaneously detect both pancreatic and biliary-tract cancers.

### Comparison of pancreato-biliary cancer with non-malignant abnormalities, or with other types of cancers

**Fig 2:**
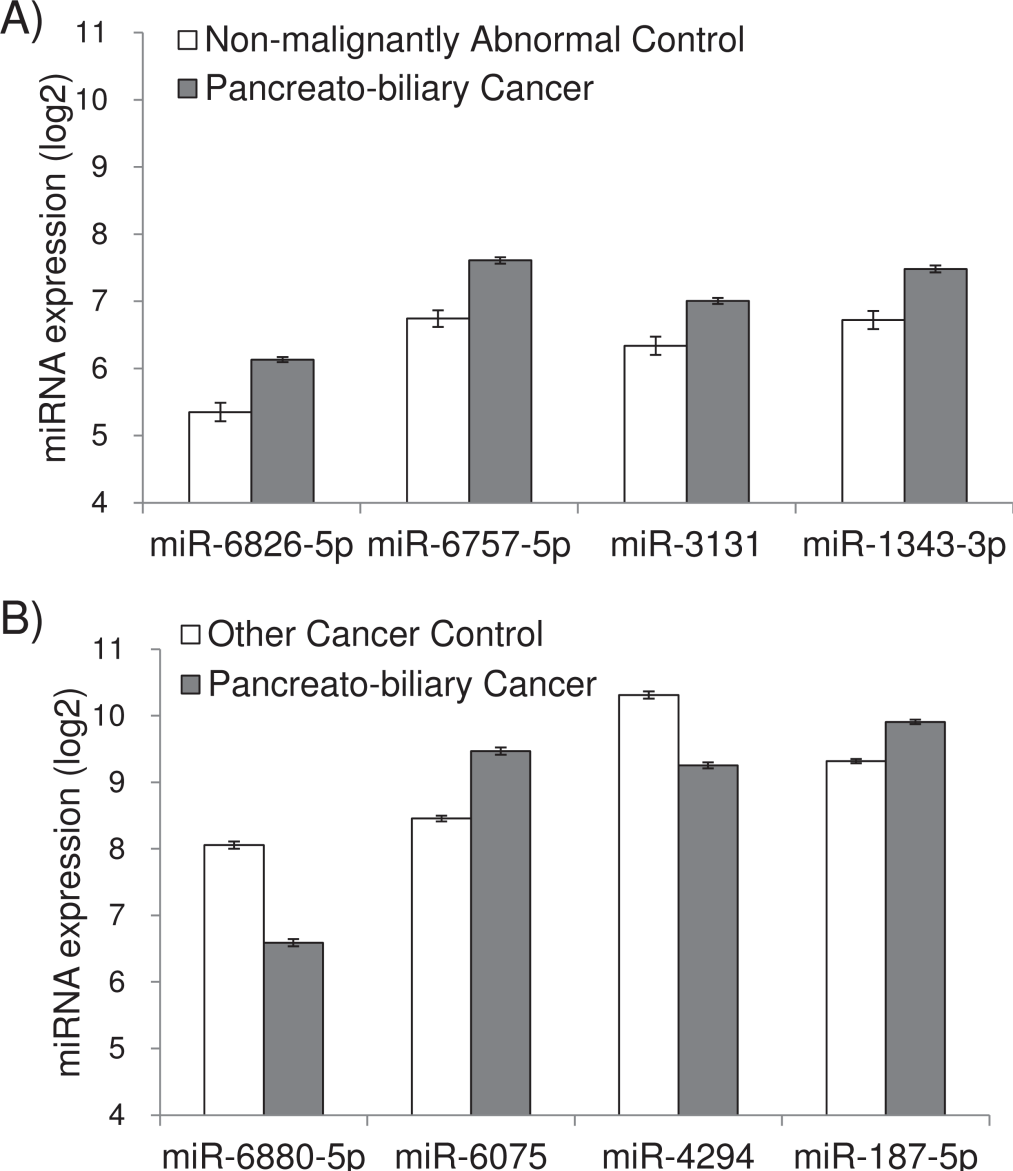
Expression signals of validated miRNAs that differentiated pancreato-biliary cancer from non-malignant abnormalities (A), or from cancers of other types (B). The error bars indicate standard error. The p-values were Bonferroni-corrected.

The ideal pancreato-biliary cancer biomarker should prove its specificity not only against healthy control but also against non-malignant disease conditions as well as other types of cancers. In order to test this thesis, we compared 198 pancreato-biliary cancer patients with 21 patients who had some abnormalities in either the pancreas or the biliary tract, but no trace of malignancy (Table 1D). We assumed that obtained miRNAs in this comparison should have represented markers for malignancy, particularly in pancreato-biliary organs. Thirty-nine miRNAs showed significantly different expressions (p<0.01) in these two groups in the training cohort, and out of those 39 miRNAs, 4 miRNAs were validated in the test cohort. These 4 miRNAs (miR-6826-5p, miR-6757-5p, miR-3131 and miR-1343-3p) were unique and have not been identified in our previous analyses of pancreato-biliary cancer in contrast with healthy control (S1 Table). Their median expression levels in the pancreato-biliary cancer group and the non-malignant abnormality group were presented in Fig. 2A. The best marker, miR-6826-5p, correctly identified not only 77.3% pancreato-biliary cancers, but also identified 85.7% non-malignant abnormalities as negative control in the test cohort. In contrast, CEA and CA19-9 correctly identified 41.4% and 70.2% pancreato-biliary cancers, and 81.0% and 81.0% non-malignant abnormalities, respectively, in the entire cohort.

Furthermore, in order to examine organ-specificity of serum miRNA markers in malignant environment, a total of 202 serum samples obtained from patients with colon, stomach, esophagus, and liver cancers (Table 1E) were analyzed, and set as another control against pancreato-biliary cancer. This comparative analysis selected 193 miRNAs in the training cohort, and among them, 120 miRNAs were validated in the test cohort. Out of 120 validated miRNAs, 26 miRNAs have been identified in the previous analysis of pancreato-biliary cancer with healthy control (S1 Table), 4 miRNAs have been identified in the analysis of pancreato-biliary cancer with non-malignant abnormalities (Fig. 2A), and the rest of 90 miRNAs were unique. The expression of 4 miRNAs (miR-6880-5p, miR-6075, miR-4294, miR-187-5p) that were significantly different between pancreato-biliary cancer and other types of cancers were presented in Fig. 2B. These four miRNAs have been also identified in our previous analysis of pancreato-biliary cancer with healthy control (S1 Table), suggesting that they are highly specific markers for pancreato-biliary cancer. In terms of diagnostic performance, miR-6880-5p correctly identified not only 66.7% pancreato-biliary cancer, but also 85.7% other cancer types as negative control in the test cohort. In contrast, CEA and CA19-9 correctly identified 41.4% and 70.2% pancreato-biliary cancers, as stated above, and 82.0% and 84.4% other cancer types in the entire cohort.

**Fig 3:**
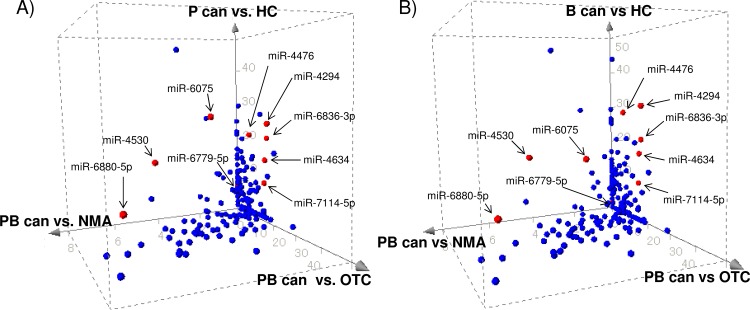
Plots of p-values in the analysis of pancreatic cancer (P can) (A) or biliary-tract cancer (B can) (B) with healthy control (HC) in z-axis, pancreato-biliary cancer (PB can) with non-malignant abnormalities (NMA) in y-axis, or pancreato-biliary cancer (PC can) with other types of cancer (OTC) in x-axis. The absolute values of the exponents of p-values on a log_10_ scale were plotted; therefore, miRNAs that were located in further outside are more statistically significant. The ten miRNA used in the diagnostic indices were specified.

The comparative analyses so far suggested that the resulting miRNA markers for pancreato-biliary cancers would largely affected by clinical characteristics of the control population. Fig. 3 shows how the significant levels of the miRNA markers that were obtained so far were distributed depending on the types of the control populations used.

### Comparison of pancreato-biliary cancers with all other clinical groups as control that included healthy individuals, and patients with other types of cancers or non-malignant abnormalities in the pancreato-biliary organs

**Fig 4:**
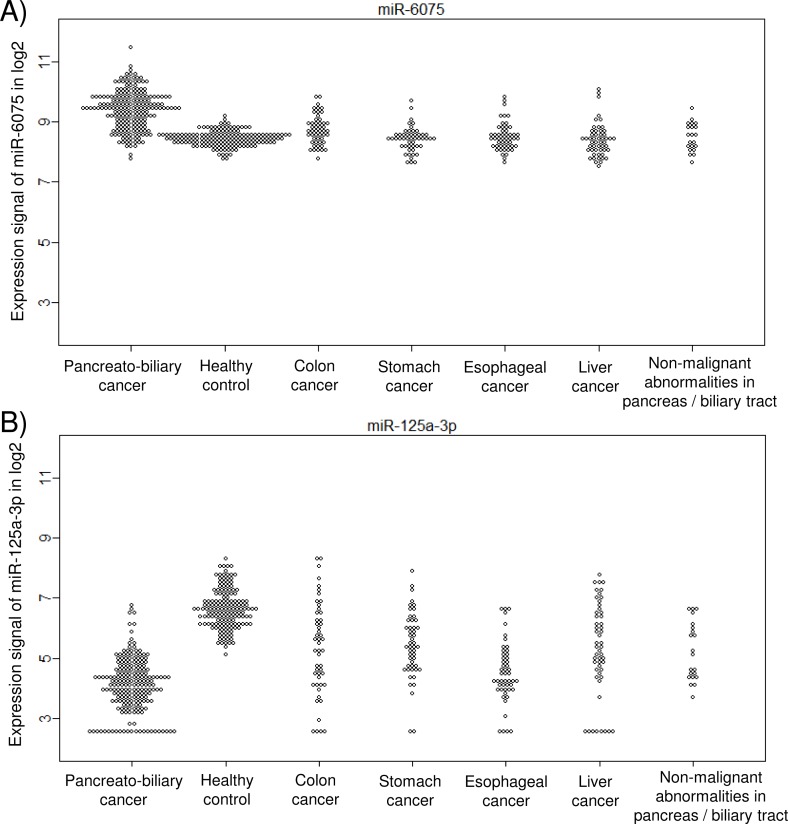
Plots of miR-6075 (A) and miR-125a-3p (B) expression signals on a log2 scale in patients with pancreato-biliary cancers, healthy control individuals, and patients with colon, stomach, esophageal, or liver cancer, or with non-malignant abnormalities in either the pancreas or the biliary tract.

In order to obtain more robust and universal markers that could identify pancreato-biliary cancer patients from clinically heterogeneous population, the supposed-control: healthy individual, patients with non-malignant abnormalities, and patients with other cancer types, were all combined, and compared with pancrato-biliary cancer patients by discriminant analysis. The analysis identified 143 miRNAs that showed differential expression in the training cohort. Among them, 98 miRNAs were statistically validated in the test cohort. Ninety-one out of those 98 validated miRNAs were already identified in the previous analyses of pancreato-biliary cancer either with healthy control, with non-malignant abnormalities, or with other types of cancer. The 10 miRNAs that showed the smallest Bonferroni-corrected p-values are listed in Table 2. All 10 markers were previously selected as markers for detecting pancreatic cancer and biliary-tract cancer against the healthy control individuals (S1 Table). The best miRNA marker for detecting pancreato-biliary cancers among the other clinical conditions was miR-6075, which was ranked fourth among the markers for detecting pancreatic cancer and seventeenth among the markers for detecting biliary-tract cancer (S1 Table) against the healthy controls. miR-6075 showed a sensitivity, a specificity, and an accuracy of 63.6%, 93.5%, and 83.2%, respectively (Fig. 4A). Similarly, the second-best marker for detecting pancreato-biliary cancers against the other clinical conditions was miR-4294, which was ranked seventh among the markers for detecting pancreatic cancer and fourth among the markers for detecting biliary-tract cancer (S1 Table). It turned out that miR-125a-3p, which was the best marker for detecting both pancreatic and biliary-tract cancers against healthy controls, also behaved similarly for the colon, stomach, liver, and esophageal cancers (Fig. 4B), resulting in a drop in its rank to fifth when the controls included the patients with non-malignant abnormalities and other cancer types (S1 Table and Table 2). This result indicated that miR-125a-3p was not a marker that is specific to pancreato-biliary cancers but was instead one that is effective for a broad range of cancer types.

**Table 2:** Top 10 validated miRNA markers that differentiated pancreato-biliary cancers from other clinical conditions including healthy controls, non-malignant abnormalities, and other types of cancers.

Rank	miRNA	Training cohort						Test cohort		
		p-value	Expression (median in log2)		Accuracy (%)	Sensitivity (%)	Specificity (%)	Accuracy (%)	Sensitivity (%)	Specificity (%)
			Pancreato-biliary cancer	All other clinical groups						
1	miR-6075	8.12E-47	9.46	8.43	85.6	78.0	89.6	83.2	63.6	93.5
2	miR-4294	7.92E-42	9.25	10.39	78.7	77.3	79.5	76.8	66.7	82.3
3	miR-6880-5p	1.02E-33	6.60	7.63	78.2	78.8	77.9	83.2	75.8	87.1
4	miR-6799-5p	1.39E-30	7.88	8.24	79.3	83.3	77.1	78.4	69.7	83.1
5	miR-125a-3p	2.01E-28	4.08	6.02	75.6	82.6	71.9	78.4	81.8	76.6
6	miR-4530	4.55E-28	8.76	9.39	77.4	80.3	75.9	77.9	71.2	81.5
7	miR-6836-3p	1.09E-27	9.25	8.70	80.8	80.3	81.1	84.7	89.4	82.3
8	miR-4634	1.68E-26	10.08	9.85	76.4	81.8	73.5	74.2	75.8	73.4
9	miR-7114-5p	5.32E-25	6.59	6.91	76.6	77.3	76.3	70.0	63.6	73.4
10	miR-4476	1.53E-23	5.79	7.01	76.9	73.5	78.7	81.6	71.2	87.1

p-values were corrected for Bonferroni.

### A diagnostic miRNA index that differentiated pancreato-biliary cancers from all other clinical conditions including healthy controls and patients with other types of cancers or non-malignant abnormalities in the pancreato-biliary organs

**Fig 5:**
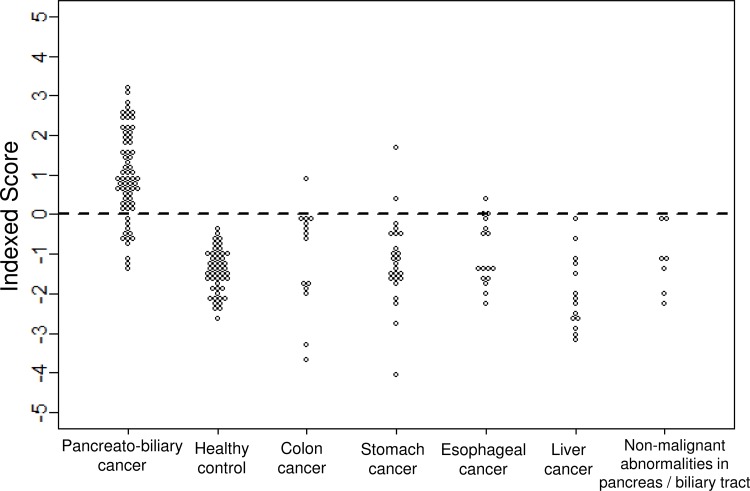
Indexed scores that gave the best discriminant performance in the test cohort with the use of four miRNAs: miR-6075, miR-6799-5p, miR-125a-3p, and miR-6836-3p.

**Fig 6:**
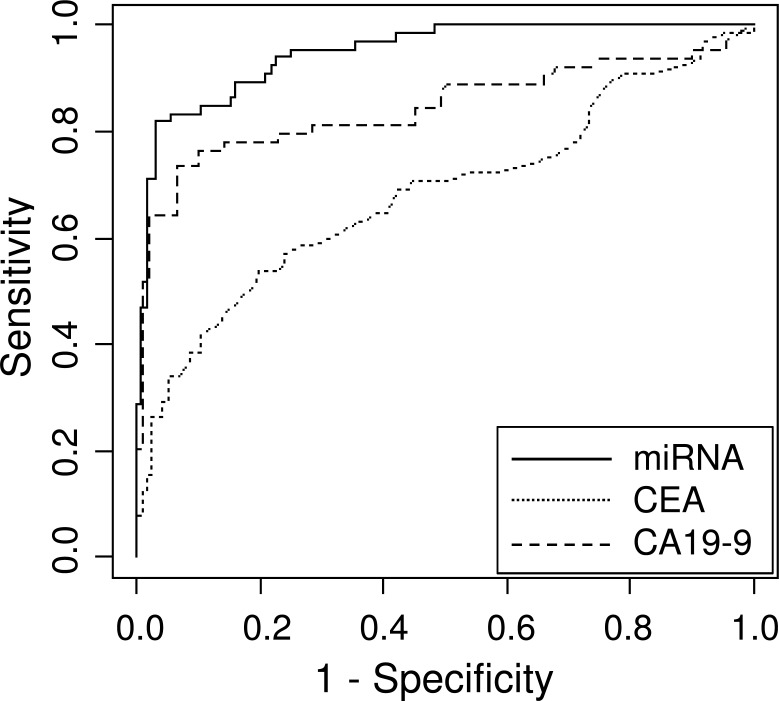
ROC analysis of a combination of four miRNAs (miR-6075, miR-6799-5p, miR-125a-3p, and miR-6836-3p) in a solid line, CEA in a dotted line, and CA19-9 in a slashed line. The AUC values were 0.949, 0.682 and 0.845 for a combination of the four miRNAs, CEA, and CA19-9, respectively. The analysis was performed in the test cohort.

According to the data above, a single serum miRNA achieved the detection of pancreatic and biliary-tract cancers with >80% accuracy. We aimed to achieve higher discriminant performance by combining multiple significant miRNAs. The 10 miRNAs (miR-6075, miR-4294, miR-6880-5p, miR-6799-5p, miR-125a-3p, miR-4530, miR-6836-3p, miR-4634, miR-7114-5p, and miR-4476) listed in Table 2 were selected as potential markers in the training cohort, and their expression signals were utilized in developing diagnostic indices. Using the Fisher’s linear discriminant analysis, all 1,023 combinations that included any one or more of the 10 miRNAs were calculated and validated in the test cohort. The miRNA combinations that showed the best accuracy with each number of miRNAs used (from 1 to 10) in this algorithm are listed in Table 3, and their discriminant functions are provided in S2 Table. It turned out that using only the single best miRNA, miR-6075, gave an accuracy of 84.7% in the detection of pancreato-biliary cancers in the test cohort; however, the accuracy increased when the number of miRNAs used in the algorithm increased, and it reached a maximum of 91.6% when four miRNAs, miR-6075, miR-6799-5p, miR-125a-3p and miR-6836-3p, were used. This combination also gave a sensitivity of 81.8% and a specificity of 96.8%. The discriminant function composed by these four miRNAs is: 1.20 x miR-6075-0.93 x miR-6799-5p—0.22 x miR-125a-3p + 0.71 x miR-6836-3p—8.55 (S2 Table), and the resultant discrimination of each clinical samples is shown in Fig. 5. The same level of accuracy was maintained when the number of miRNAs used in the algorithm was increased up to eight, and it then started to decrease when the miRNAs ranked ninth (accuracy 90.0%) and tenth (accuracy 88.9%) were added. Beside accuracy obtained by Fisher’s linear discriminatory analysis, ROC analysis gave slightly different result (Table 3); the AUC value started as 0.930 with the single best miRNA, miR-6075, and gradually increased with some fluctuation, and reached the maximum of 0.967 with a combination of the nine miRNAs (miR-6075, miR-4294, miR-6880-5p, miR-6799-5p, miR-125a-3p, miR-4530, miR-6836-3p, miR-7114-5p, and miR-4476). Both analyses indicated that the diagnostic performance would reach to saturation when combinations of the ten miRNAs were used in the algorithm. The AUC of CEA and CA19-9 was 0.682 and 0.845, respectively, in the same test cohort (Fig. 6). No correlation was found between the expression of either CEA or CA19-9 and the expression of any of these ten miRNA markers used in the diagnostic indices.

**Table 3:** Combinations of miRNAs that showed the highest accuracy for each number of miRNAs used (one to ten) and their diagnostic performance in the test cohort.

Number of miRNAs used	Combinations of miRNAs	Accuracy (%)	Sensitivity (%)	Specificity (%)	AUC
1	miR-6836-3p	84.7	89.4	82.3	0.930
2	miR-6075, miR-6836-3p	89.5	75.8	96.8	0.941
3	miR-6075, miR-6836-3p, miR-4476	91.1	78.8	97.6	0.949
4	miR-6075, miR-6799-5p, miR-125a-3p, miR-6836-3p	91.6	81.8	96.8	0.949
5	miR-6075, miR-4294, miR-6799-5p, miR-125a-3p, miR-6836-3p	91.6	81.8	96.8	0.948
	miR-6075, miR-6799-5p, miR-125a-3p, miR-4530, miR-6836-3p	91.6	81.8	96.8	0.950
6	miR-6075, miR-4294, miR-6799-5p, miR-125a-3p, miR-4530, miR-6836-3p	91.6	81.8	96.8	0.948
	miR-6075, miR-6880-5p, miR-6799-5p, miR-125a-3p, miR-6836-3p, miR-4476	91.6	80.3	97.6	0.955
7	miR-6075, miR-6880-5p, miR-6799-5p, miR-125a-3p, miR-4530, miR-6836-3p, miR-4476	91.6	80.3	97.6	0.953
8	miR-6075, miR-4294, miR-6880-5p, miR-6799-5p, miR-125a-3p, miR-4530, miR-6836-3p, miR-4476	91.6	80.3	97.6	0.953
9	miR-6075, miR-4294, miR-6880-5p, miR-6799-5p, miR-125a-3p, miR-4530, miR-6836-3p, miR-7114-5p, miR-4476	90.0	80.3	95.2	0.967
	miR-6075, miR-4294, miR-6880-5p, miR-6799-5p, miR-125a-3p, miR-6836-3p, miR-4634, miR-7114-5p, miR-4476	90.0	78.8	96.0	0.965
10	miR-6075, miR-4294, miR-6880-5p, miR-6799-5p, miR-125a-3p, miR-4530, miR-6836-3p, miR-4634, miR-7114-5p, miR-4476	88.9	78.8	94.4	0.966

We then focused on the algorithm that used the combination of four miRNAs: miR-6075, miR-6799-5p, miR-125a-3p, and miR-6836-3p, which was the combination with the least number of miRNAs that achieved the best accuracy calculated (Table 3). The number of positive and negative samples in each clinical group and cohort based on that miRNA discriminant, CEA or CA19-9 test are shown in Table 4. The discriminant index identified pancreato-biliary cancers with equivalent diagnostic power regardless of the tumor operability or location. Five (one ypStage I, 1 ypStage IIB, 2 pStage II and 1 cStage III) of 7 operable pancreatic cancers and 17 of 22 operable biliary-tract cancers in the test cohort were correctly detected by the index. When the entire cohort was included in the analysis, the index detected 18 of 21 (85.7%) operable pancreatic cancer and 38 of 48 (79.2%) operable biliary-tract cancer. It should be noted, however, that the discriminant performance of the index was not correlated with the tumor stage. Among the 22 CA19-9-negative patients with either pancreatic cancer or biliary-tract cancer in the test cohort, 20 (83.3%) were correctly detected by the discriminant index. Finally, the diagnostic power of this index was not affected by tumor location of either pancreatic or biliary-tract cancer.

**Table 4:** The number of positive and negative samples by CEA, CA19-9, and the diagnostic index* tests in each clinical group.

	Clinical group	Stage or operability	CEA (median)		CA19-9		Diagnostic Index*	
			Positive	Negative	Positive	Negative	Positive	Negative
Training cohort (381)	Healthy control (102)	-	4	98	3	99	0	102
	Pancreatic cancer (71)	pStage I	0	0	0	0	0	0
		pStage II	2	11	9	4	12	1
		cStage III	9	12	16	5	19	2
		cStage IV	25	12	30	7	34	3
	Biliary-tract cancer (61)	Operable	6	20	14	12	21	5
		Inoperable**	14	20	28	5	27	8
	Non-malignant abnormalities in pancreas or biliary tract (14)	-	2	12	3	11	6	8
	Colon cancer (36)	-	12	24	6	30	11	25
	Stomach cancer (25)	-	2	23	4	21	2	23
	Esophageal cancer (34)**	-	5	28	-	-	9	25
	Liver cancer (38)**	-	4	17	3	5	5	33
Test cohort (176)	Healthy control (48)	-	2	46	0	48	0	48
	Pancreatic cancer (29)	pStageI	1	0	1	0	1	0
		pStageII	1	4	3	2	3	2
		cStageIII	3	3	5	1	5	1
		cStageIV	11	6	13	4	15	2
	Biliary-tract cancer (37)	Operable**	3	18	10	10	17	5
		Inoperable	7	8	10	5	13	2
	Non-malignant abnormalities in pancreas or biliary tract (7)	-	2	5	1	6	0	7
	Colon cancer (14)	-	4	10	3	11	1	13
	Stomach cancer (25)	-	1	24	3	22	2	23
	Esophageal cancer (16)**	-	2	14	-	-	1	15
	Liver cancer (14)**	-	3	10	0	4	0	14

*The diagnostic index used the following four miRNAs: miR-6075, miR-6799-5p, miR-125a-3p, and miR-6836-3p.

**CA19-9 and/or CEA scores were not available in some cases of biliary-tract cancer, esophageal cancer, and liver cancer.

On the other hand, 5 of 29 pancreatic cancers and 7 of 37 biliary-tract cancers in the test cohort gave false-negative scores by the miRNA index. These false-negative cases did not show any partiality in their clinical characteristics; between the false-negative and the true-positive cases, there was no significant difference in the patients’ age (p = 0.111), serum CA19-9 (p = 0.581) or CEA (p = 0.063) concentrations nor was there a difference in the histological location of the tumor or the sites of distant metastasis. Similarly, 4 of 124 control samples gave false-positive scores by the miRNA index. The false-positive samples varied in their clinical status (One colon cancer, 1 esophageal cancer, 2 stomach cancers) and did not show any particular clinical characteristics that might indicate any bias. There was one non-malignant abnormality case that was initially diagnosed as chronic pancreatitis, but after 3-year follow-up, pancreatic cancer was found. This case was designated in the training cohort, and was negative by both CEA (1.2 ng/mL) and CA19-9 (10 U/mL) test, but positive by our diagnostic index.

These results suggest that the diagnostic index is able to detect pancreato-biliary cancers at various stages and histological locations, and discriminates not only against healthy individuals but also against individuals with non-malignant abnormalities in pancreato-biliary organs as well as those with other types of cancers.

### Validation of the miRNA expression in normal and cancerous tissues of the pancreas

The expression of the miRNAs selected for the diagnostic indices was validated in the FFPE tissue samples obtained from 10 patients with pancreatic cancer. Paired samples of cancerous tissue and the surrounding normal tissue were separately obtained from the same patients by macroscopic-dissection. The expression of those ten miRNAs that were used in the discriminant index was quite strong in the pancreatic cancer tissue (all within the top 10% of miRNAs in terms of the median expression signals); however, they did not show statistical significance in differentiating the cancerous tissue from the normal surrounding tissue (Table 5), indicating that miRNA markers obtained from serum samples may not comparable with those obtained from corresponding tissue samples.

**Table 5:** Expression of the ten miRNAs used in the discriminant indices in paired malignant and normal tissues obtained from the same patients with pancreatic cancer (n = 10).

miRNA	Expression Signal (Median)		p-value
	Normal (n = 10)	Tumor (n = 10)	
miR-6075	11.3	11.2	5.9.E-01
miR-4294	8.8	9.0	6.2.E-01
miR-6880-5p	8.4	8.5	8.6.E-01
miR-6799-5p	10.7	10.0	8.3.E-02
miR-125a-3p	10.1	9.8	1.5.E-01
miR-4530	12.9	12.7	1.0.E-01
miR-6836-3p	7.6	7.3	1.9.E-01
miR-4634	8.7	8.5	1.2.E+00
miR-7114-5p	10.0	9.9	2.2.E+00
miR-4476	8.0	8.0	8.3.E-01

## Discussion

Despite the desperate need for the early detection of pancreatic cancer, there is no simple, least-invasive screening method. Pancreatic cancer has such a low prevalence in general population that it may be difficult even for accurate diagnostic tests to achieve sufficient positive-predictive values in population-wide screening [13]. Since imaging tests still have their limitations in diagnosis of pancreatic cancers, particularly of those at early stages [2, 14], development of novel blood marker tests for pancreatic cancer is urgently needed.

The current blood biomarkers CEA and CA19-9 have been prevalently used for post-operation monitoring, but their poor specificity for non-malignant diseases and other cancers as well as their poor sensitivities to early cancers prevent them from being widely used in screening systems. In this study, CEA and CA19-9 failed to detect not only the early stage of pancreatic and biliary-tract cancers but also progressed cancers. A simple blood screening-test alternative to those protein markers is necessary for pancreato-biliary cancers.

Several prior studies have shown the value of serum miRNAs in the detection of several kinds of cancers [5–9, 15, 16]. A number of new cancer-related miRNAs have been recently identified, and most of them have never been examined in pancreatic cancer. In this study, using highly sensitive microarrays that permitted the simultaneous analysis of more than 2,500 miRNAs that were recently updated in the miRBase (release 20), we examined the expression profiles of comprehensive serum miRNAs in patients with pancreato-biliary cancers. In addition to 100 patients with pancreatic cancer and 98 patients with biliary-tract cancer, we examined 150 healthy control individuals, 21 patients with non-malignant abnormalities in either the pancreas or the biliary tract, and 202 patients with other types of cancers (esophagus, stomach, liver, and colon) to determine the discriminant performance in various clinical settings, allowing us to study the markers in the largest and most comprehensive clinical cohort that has yet been attempted. Especially, the study setting with the control cohort that is consisted of diverse populations is critical when envisaging the implementation of biomakers in real clinical setting. At first, we tried to discover serum miRNA markers for pancreatic cancer, and we obtained 81 miRNAs in a comparative analysis with healthy controls. However, 55 of those miRNAs (67.9%) were also markers that discriminated between biliary-tract cancers and healthy control samples. That finding suggested that the exclusive identification of pancreatic cancers and biliary-tract cancers, respectively, by serum miRNA was so difficult that we decided to find markers that detect pancreatic and biliary-tract cancers simultaneously. Those results had led to our concern that any cancer type would show similar miRNA expression profiles in the serum, and that we would be unable to discriminate other cancer types from pancreato-biliary malignancies. We therefore additionally prepared samples of other types of digestive-tract cancers. Furthermore, we prepared samples from patients with non-malignant abnormalities, because CA19-9, the most frequently used biomarker for pancreatic cancer, is known to be expressed in non-malignant abnormalities such as inflammation, leading to poor specificity toward that clinical group.

In this study, the best single marker achieved an accuracy of 84.7% in detecting pancreato-biliary cancers. By combining multiple significant miRNAs, we had thought to attain higher diagnostic performance as previously demonstrated [17, 18]. Utilizing the 10 most significant miRNAs deduced from the above comparative analyses, diagnostic indices were developed, and their discriminant performance was validated in the test cohort. By combining 8 miRNAs (miR-6075, miR-4294, miR-6880-5p, miR-6799-5p, miR-125a-3p, miR-4530, miR-6836-3p, and miR-4476) out of the 10 miRNA selected, we finally achieved the best discriminant performance with a sensitivity of 80.3%, a specificity of 97.6%, and an accuracy of 91.6% in detecting pancreato-biliary cancers among healthy control, non-malignant abnormalities or other types of cancers. When more miRNAs (in this case, nine or more) were integrated into the diagnostic indices, the discriminant performance started to decline, possibly due to the pick-up of noise features. Such a phenomenon is known as the “curse of dimensionality” in algorithm development [19].

We further validated the detailed result of the discriminant function composed of four miRNAs (the combination with the least number of miRNAs). The detailed validation of the discriminant performance revealed the clinical value of the diagnostic index; the index identified the early phase of pancreato-biliary cancers that were surgically resectable. Because the current best treatment for pancreato-biliary cancers is the resection of tumors while the tumors are still localized in the organs, a diagnostic index capable of detecting small resectable tumors is promising. Furthermore, this index was able to detect any histological type of tumors, some of which (e.g., tumors located in the pancreatic tail or the intrahepatic bile duct) could be difficult to detect by image screening if not well attended. This index which utilizes blood miRNA markers could be used beforehand to provide a rationale to perform more costly and/or invasive screening tests such as a CT scan or endoscopic ultrasound.

Although we found that a combination of serum miRNAs could become a specific marker for pancreato-biliary cancers, the biological roles of those serum miRNAs are under discussion. Nine out of the ten miRNAs used for the diagnostic indices had an ID name higher than 1,000, indicating that they were discovered relatively recently. We were therefore not able to find many references about those miRNAs except for miR-125a-3p. Serum miR-125a-3p has been reported to show different expression profiles between patients with pancreatic cancers and healthy individuals [5, 7]. In the current study, we also found that miR-125a-3p is a strong candidate for the detection of pancreato-biliary cancers in the independent analysis. However, the integrated analysis showed that miR-125a-3p was differentially expressed in sera from patients with other digestive-tract cancers as well, indicating that that miRNA is not a cancer type-specific marker but is instead a broad marker similar to CEA or CA19-9.

One of the mRNAs targeted by miR-125a-3p is MYST/Esa1-associated factor 6 (MEAF6), which encodes a nucleic protein that is associated with transcriptional activators. It is reported that MEAF6 binds with NuF4 complex, which acetylates histone H4 [20]; and this NuF4 complex is known to interact with the oncogenic transcriptional regulator c-Myc [21]. It could be hypothesized that the decreased miR-125a-3p expression leads to a reduction of the repressing effect on the above oncogenes, promoting carcinogenesis.

It is hypothesized that cancer cells secrete exosomes that encompass various molecular informants including proteins, DNA, and miRNAs into the blood stream and send them to distant organs in order to cultivate a new environment for future metastasis [15, 16]. According to this hypothesis, sera from patients with cancer should contain more miRNAs than those from healthy individuals; however, we did not observe such a phenomenon based on the overall expression-signal intensity obtained from the same 300-μL serum samples. In fact, the expression levels of seven out of ten miRNA markers (miR-6075, miR-4294, miR-6880-5p, miR-6799-5p, miR-125a-3p, miR-4530, miR-6836-3p, miR-4634, miR-7114-5p, miR-4476) used for the diagnostic indices were decreased in the serum samples from the patients with cancer compared with those from the healthy control individuals. Therefore, other biological cascades should also be considered for the secretion of circulating miRNAs.

Another enigma is the discordance of miRNA expression profiles in the serum and in the tissues. The 10 miRNAs that were significantly expressed in the sera of the patients with pancreato-biliary cancers did not show similar significant increases in the FFPE cancerous tissues compared with the surrounding normal tissues. This observation is again not in accordance with the tumor-secreted exosome theory. We think that the underlying mechanism of circulating miRNAs is more complex than previously speculated. Furthermore, we have preliminary data indicating that expression profiles of those miRNAs that were especially derived from clinical samples such as blood or FFPE tend to fluctuate depending on the assay method (microarray, PCR, sequencer) used (data not shown). In short, a number of studies remain to be performed to elucidate the biological significance of the miRNAs that we found to be involved in pancreatic and biliary-tract carcinogenesis. Until such a mechanism is clarified, the serum miRNA markers for pancreato-biliary cancers should not be confused with the tissue miRNA markers.

In conclusion, our results provide strong data showing that serum miRNAs, particularly diagnostic index conducted by the combination of several predictive miRNAs, can detect patients with pancreatic and biliary-tract cancers against a background of those who are healthy, have other non-malignant diseases or have other types of cancer. We believe that detecting pancreato-biliary cancers using peripheral blood, which is relatively easy to obtain from most subjects, is clinically useful, particularly as a first screening test. Such a test will provide additional information to those who could be at risk and, if necessary, encourage them to take more costly, sometimes invasive, imaging tests. We hope that such a test will lead to the early detection of these cancers and to improvement in the currently devastating survival rate.

## Supporting Information

S1 Table Validated miRNA markers that differentiate between patients with pancreatic cancer and healthy control individuals (A) or patients with biliary-tract cancer and healthy control individuals (B). (DOCX)

S2 Table Best discriminate functions for each possible number of miRNAs used in the test cohort. (DOCX)
