# Supplementary material for: MicroRNA Markers for the Diagnosis of Pancreatic and Biliary-Tract Cancers
Source: PLoS One. 2015 Feb 23;10(2):e0118220. doi: 10.1371/journal.pone.0118220 (PMC4338196; doi:10.1371/journal.pone.0118220)
Supplement: S1 Table — (DOCX) [file pone.0118220.s001.docx]

SUPPORTING INFORMATION

S1 Table. Validated miRNA markers that differentiate between patients with pancreatic cancer and healthy control individuals (A) or patients with biliary-tract cancer and healthy control individuals (B).

| A) Pancreatic cancer vs. healthy control | | | | | | | | | | | | | | |
| --- | --- | --- | --- | --- | --- | --- | --- | --- | --- | --- | --- | --- | --- | --- |
| Rank | miRNA | Training cohort | | | | | | | | Test cohort | | | | |
|  |  | Expression (median in log2) | | | | p-value | Accuracy (%) | Sensitivity (%) | Specificity (%) | p-value | | Accuracy (%) | Sensitivity (%) | Specificity (%) |
|  |  | Healthy control | | Pancreatic cancer | |  |  |  |  |  |  |  |  |  |
| 1 | miR-125a-3p | 6.69 | | 4.35 | | 1.76E-48 | 97.0 | 94.0 | 99.0 | 1.33E-21 | | 95.2 | 90.9 | 98.0 |
| 2 | miR-6893-5p | 8.66 | | 7.22 | | 6.19E-46 | 96.4 | 95.5 | 97.0 | 1.93E-17 | | 92.8 | 87.9 | 96.0 |
| 3 | miR-204-3p | 13.26 | | 11.66 | | 1.60E-30 | 89.2 | 79.1 | 96.0 | 4.64E-11 | | 79.5 | 78.8 | 80.0 |
| 4 | miR-6075 | 8.32 | | 9.37 | | 1.69E-29 | 90.4 | 79.1 | 98.0 | 1.74E-20 | | 96.4 | 93.9 | 98.0 |
| 5 | miR-1469 | 10.06 | | 10.58 | | 1.59E-28 | 89.2 | 80.6 | 95.0 | 4.67E-13 | | 88.0 | 84.8 | 90.0 |
| 6 | miR-6820-5p | 7.38 | | 6.67 | | 1.56E-27 | 88.0 | 88.1 | 88.0 | 1.26E-06 | | 78.3 | 69.7 | 84.0 |
| 7 | miR-4294 | 10.36 | | 9.44 | | 3.21E-27 | 87.4 | 79.1 | 93.0 | 6.80E-15 | | 88.0 | 90.9 | 86.0 |
| 8 | miR-575 | 6.16 | | 4.78 | | 1.62E-26 | 86.2 | 82.1 | 89.0 | 6.82E-12 | | 88.0 | 87.9 | 88.0 |
| 9 | miR-6729-5p | 12.49 | | 12.74 | | 6.52E-26 | 88.0 | 85.1 | 90.0 | 1.33E-11 | | 84.3 | 81.8 | 86.0 |
| 10 | miR-4476 | 7.34 | | 6.01 | | 3.85E-23 | 85.0 | 79.1 | 89.0 | 1.98E-10 | | 80.7 | 72.7 | 86.0 |
| 11 | miR-150-3p | 6.92 | | 6.04 | | 7.47E-23 | 82.6 | 83.6 | 82.0 | 2.94E-08 | | 73.5 | 84.8 | 66.0 |
| 12 | miR-6836-3p | 8.61 | | 9.20 | | 2.37E-22 | 91.0 | 88.1 | 93.0 | 1.70E-06 | | 89.2 | 90.9 | 88.0 |
| 13 | miR-6765-3p | 9.01 | | 7.75 | | 4.52E-22 | 86.8 | 82.1 | 90.0 | 4.72E-09 | | 81.9 | 69.7 | 90.0 |
| 14 | miR-423-5p | 7.43 | | 6.59 | | 4.83E-21 | 80.8 | 74.6 | 85.0 | 7.07E-04 | | 74.7 | 72.7 | 76.0 |
| 15 | miR-6799-5p | 8.18 | | 7.80 | | 4.64E-19 | 80.2 | 82.1 | 79.0 | 5.23E-08 | | 84.3 | 87.9 | 82.0 |
| 16 | miR-4530 | 9.49 | | 8.80 | | 8.49E-19 | 80.2 | 80.6 | 80.0 | 4.03E-07 | | 75.9 | 84.8 | 70.0 |
| 17 | miR-7641 | 7.84 | | 6.35 | | 1.16E-17 | 79.6 | 73.1 | 84.0 | 1.39E-06 | | 79.5 | 84.8 | 76.0 |
| 18 | miR-4454 | 11.85 | | 11.06 | | 1.25E-17 | 81.9 | 75.8 | 86.0 | 2.08E-07 | | 81.9 | 72.7 | 88.0 |
| 19 | miR-8073 | 6.12 | | 6.79 | | 2.59E-17 | 83.2 | 74.6 | 89.0 | 5.05E-06 | | 83.1 | 72.7 | 90.0 |
| 20 | miR-615-5p | 6.65 | | 6.12 | | 2.90E-17 | 83.2 | 88.1 | 80.0 | 5.50E-08 | | 77.1 | 78.8 | 76.0 |
| 21 | miR-4450 | 6.09 | | 4.61 | | 8.38E-17 | 82.6 | 70.1 | 91.0 | 5.07E-09 | | 80.7 | 78.8 | 82.0 |
| 22 | miR-4634 | 9.73 | | 9.98 | | 2.49E-16 | 79.6 | 83.6 | 77.0 | 9.37E-05 | | 73.5 | 75.8 | 72.0 |
| 23 | miR-4792 | 6.37 | | 7.33 | | 6.53E-16 | 82.0 | 79.1 | 84.0 | 5.28E-12 | | 85.5 | 84.8 | 86.0 |
| 24 | miR-665 | 6.98 | | 7.51 | | 8.11E-16 | 80.2 | 74.6 | 84.0 | 4.29E-08 | | 81.9 | 66.7 | 92.0 |
| 25 | miR-7975 | 10.14 | | 9.34 | | 8.38E-15 | 77.8 | 70.1 | 83.0 | 1.40E-04 | | 75.9 | 63.6 | 84.0 |
| 26 | miR-7109-5p | 7.51 | | 7.20 | | 3.13E-14 | 77.2 | 77.6 | 77.0 | 4.70E-05 | | 77.1 | 84.8 | 72.0 |
| 27 | miR-6789-5p | 9.78 | | 10.09 | | 4.53E-13 | 80.2 | 80.6 | 80.0 | 9.49E-09 | | 81.9 | 81.8 | 82.0 |
| 28 | miR-4497 | 12.95 | | 12.27 | | 5.21E-13 | 74.9 | 74.6 | 75.0 | 6.51E-03 | | 64.6 | 56.2 | 70.0 |
| 29 | miR-6880-5p | 7.38 | | 6.72 | | 5.29E-13 | 77.2 | 73.1 | 80.0 | 6.99E-10 | | 78.3 | 84.8 | 74.0 |
| 30 | miR-6877-5p | 7.26 | | 6.94 | | 5.66E-13 | 80.2 | 76.1 | 83.0 | 7.81E-12 | | 84.3 | 90.9 | 80.0 |
| 31 | miR-7977 | 10.00 | | 9.31 | | 6.23E-13 | 77.2 | 68.7 | 83.0 | 4.82E-05 | | 75.9 | 57.6 | 88.0 |
| 32 | miR-4734 | 11.80 | | 12.11 | | 6.56E-13 | 76.0 | 79.1 | 74.0 | 7.66E-04 | | 61.4 | 63.6 | 60.0 |
| 33 | miR-8089 | 6.62 | | 6.23 | | 8.16E-13 | 74.9 | 73.1 | 76.0 | 1.63E-04 | | 71.1 | 69.7 | 72.0 |
| 34 | miR-6085 | 10.48 | | 10.24 | | 4.16E-12 | 75.4 | 65.7 | 82.0 | 3.16E-06 | | 69.9 | 75.8 | 66.0 |
| 35 | miR-5585-3p | 5.36 | | 6.19 | | 9.67E-12 | 80.2 | 79.1 | 81.0 | 1.00E-08 | | 85.5 | 75.8 | 92.0 |
| 36 | miR-4651 | 11.00 | | 10.68 | | 1.48E-11 | 76.0 | 70.1 | 80.0 | 1.33E-06 | | 77.1 | 69.7 | 82.0 |
| 37 | miR-4433-3p | 7.12 | | 7.61 | | 6.32E-11 | 77.2 | 82.1 | 74.0 | 8.89E-06 | | 75.9 | 75.8 | 76.0 |
| 38 | miR-564 | 6.18 | | 5.06 | | 1.13E-10 | 77.2 | 64.2 | 86.0 | 1.01E-06 | | 79.5 | 72.7 | 84.0 |
| 39 | miR-1231 | 6.36 | | 6.83 | | 1.51E-10 | 76.0 | 71.6 | 79.0 | 4.15E-13 | | 81.9 | 97.0 | 72.0 |
| 40 | miR-1238-5p | 6.31 | | 6.72 | | 4.82E-10 | 81.4 | 70.1 | 89.0 | 3.41E-04 | | 74.7 | 66.7 | 80.0 |
| 41 | miR-7114-5p | 6.92 | | 6.61 | | 5.49E-10 | 76.6 | 73.1 | 79.0 | 3.11E-05 | | 77.1 | 72.7 | 80.0 |
| 42 | miR-8069 | 12.74 | | 12.95 | | 7.14E-10 | 76.0 | 68.7 | 81.0 | 1.64E-04 | | 78.3 | 72.7 | 82.0 |
| 43 | miR-619-5p | 7.33 | | 8.11 | | 2.10E-09 | 76.6 | 68.7 | 82.0 | 2.77E-06 | | 80.7 | 60.6 | 94.0 |
| 44 | miR-3188 | 5.82 | | 6.26 | | 5.00E-09 | 77.2 | 76.1 | 78.0 | 1.47E-09 | | 80.7 | 84.8 | 78.0 |
| 45 | miR-6741-5p | 6.96 | | 6.67 | | 5.70E-09 | 70.1 | 64.2 | 74.0 | 2.02E-05 | | 72.3 | 72.7 | 72.0 |
| 46 | miR-6125 | 11.85 | | 12.11 | | 7.27E-09 | 73.1 | 71.6 | 74.0 | 2.07E-07 | | 80.7 | 87.9 | 76.0 |
| 47 | miR-6805-5p | 11.34 | | 11.48 | | 8.42E-09 | 71.3 | 65.7 | 75.0 | 2.16E-03 | | 71.1 | 63.6 | 76.0 |
| 48 | miR-1246 | 7.89 | | 8.89 | | 1.95E-08 | 80.8 | 79.1 | 82.0 | 2.51E-04 | | 77.1 | 66.7 | 84.0 |
| 49 | miR-6875-5p | 8.77 | | 9.04 | | 2.68E-08 | 69.5 | 65.7 | 72.0 | 1.76E-04 | | 76.8 | 78.1 | 76.0 |
| 50 | miR-4736 | 5.73 | | 6.10 | | 6.78E-08 | 70.1 | 67.2 | 72.0 | 2.43E-04 | | 75.9 | 63.6 | 84.0 |
| 51 | miR-4732-5p | 6.28 | | 7.09 | | 8.37E-08 | 77.8 | 70.1 | 83.0 | 4.10E-04 | | 74.7 | 63.6 | 82.0 |
| 52 | miR-6724-5p | 9.81 | | 10.06 | | 8.75E-08 | 70.7 | 70.1 | 71.0 | 1.30E-06 | | 73.5 | 84.8 | 66.0 |
| 53 | miR-7107-5p | 7.91 | | 7.64 | | 9.46E-08 | 74.9 | 74.6 | 75.0 | 6.44E-03 | | 65.1 | 66.7 | 64.0 |
| 54 | miR-3622a-5p | 6.05 | | 5.64 | | 1.34E-07 | 75.4 | 59.7 | 86.0 | 8.55E-06 | | 79.5 | 75.8 | 82.0 |
| 55 | miR-6726-5p | 10.02 | | 9.67 | | 2.38E-07 | 74.9 | 61.2 | 84.0 | 8.21E-04 | | 72.3 | 63.6 | 78.0 |
| 56 | miR-3185 | 6.69 | | 7.38 | | 3.00E-07 | 73.7 | 70.1 | 76.0 | 1.15E-07 | | 77.1 | 78.8 | 76.0 |
| 57 | miR-1273g-3p | 7.57 | | 7.93 | | 6.70E-07 | 70.7 | 61.2 | 77.0 | 6.31E-03 | | 69.9 | 51.5 | 82.0 |
| 58 | miR-6779-5p | 7.17 | | 6.96 | | 1.12E-06 | 73.1 | 74.6 | 72.0 | 4.09E-04 | | 69.9 | 78.8 | 64.0 |
| 59 | miR-4723-5p | 8.95 | | 8.58 | | 1.27E-06 | 74.3 | 73.1 | 75.0 | 6.30E-09 | | 75.9 | 87.9 | 68.0 |
| 60 | miR-6850-5p | 11.25 | | 11.44 | | 1.62E-06 | 70.7 | 67.2 | 73.0 | 1.01E-04 | | 69.9 | 78.8 | 64.0 |
| 61 | miR-602 | 6.18 | | 6.62 | | 2.00E-06 | 71.9 | 74.6 | 70.0 | 1.15E-05 | | 69.9 | 69.7 | 70.0 |
| 62 | miR-8072 | 12.21 | | 12.44 | | 5.11E-06 | 71.9 | 77.6 | 68.0 | 2.12E-04 | | 73.5 | 81.8 | 68.0 |
| 63 | miR-1290 | 5.33 | | 6.32 | | 7.86E-06 | 71.3 | 71.6 | 71.0 | 4.60E-04 | | 72.3 | 66.7 | 76.0 |
| 64 | miR-4486 | 6.96 | | 7.31 | | 8.25E-06 | 69.5 | 64.2 | 73.0 | 7.73E-06 | | 75.9 | 72.7 | 78.0 |
| 65 | miR-7106-5p | 6.13 | | 5.70 | | 3.25E-05 | 72.5 | 71.6 | 73.0 | 4.77E-04 | | 66.3 | 69.7 | 64.0 |
| 66 | miR-6780b-5p | 8.69 | | 8.98 | | 4.26E-05 | 69.5 | 70.1 | 69.0 | 3.92E-04 | | 74.1 | 64.5 | 80.0 |
| 67 | miR-6090 | 12.92 | | 13.10 | | 4.85E-05 | 66.5 | 71.6 | 63.0 | 3.07E-03 | | 65.1 | 78.8 | 56.0 |
| 68 | miR-187-5p | 10.09 | | 9.81 | | 1.08E-04 | 65.9 | 70.1 | 63.0 | 2.21E-04 | | 71.1 | 78.8 | 66.0 |
| 69 | miR-4534 | 6.81 | | 6.46 | | 1.32E-04 | 73.1 | 67.2 | 77.0 | 7.97E-06 | | 73.5 | 84.8 | 66.0 |
| 70 | miR-4449 | 6.34 | | 6.56 | | 1.65E-04 | 67.7 | 67.2 | 68.0 | 4.38E-06 | | 77.1 | 84.8 | 72.0 |
| 71 | miR-5195-3p | 7.03 | | 6.64 | | 1.73E-04 | 65.7 | 63.6 | 67.0 | 2.09E-04 | | 77.1 | 84.8 | 72.0 |
| 72 | miR-1202 | 6.68 | | 6.41 | | 2.15E-04 | 70.1 | 73.1 | 68.0 | 3.88E-03 | | 71.1 | 69.7 | 72.0 |
| 73 | miR-1908-5p | 11.34 | | 11.60 | | 4.15E-04 | 68.3 | 59.7 | 74.0 | 5.00E-04 | | 75.9 | 75.8 | 76.0 |
| 74 | miR-4467 | 9.49 | | 9.85 | | 7.15E-04 | 68.3 | 58.2 | 75.0 | 4.33E-06 | | 78.3 | 87.9 | 72.0 |
| 75 | miR-4281 | 11.66 | | 11.48 | | 8.53E-04 | 67.7 | 62.7 | 71.0 | 3.16E-03 | | 72.3 | 69.7 | 74.0 |
| 76 | miR-3162-5p | 7.46 | | 7.20 | | 1.30E-03 | 67.7 | 68.7 | 67.0 | 1.91E-05 | | 77.1 | 78.8 | 76.0 |
| 77 | miR-371a-5p | 7.49 | | 7.26 | | 1.48E-03 | 67.1 | 70.1 | 65.0 | 4.17E-03 | | 71.1 | 81.8 | 64.0 |
| 78 | miR-1227-5p | 9.42 | | 9.54 | | 1.60E-03 | 65.9 | 59.7 | 70.0 | 5.29E-03 | | 67.5 | 66.7 | 68.0 |
| 79 | miR-6722-3p | 8.45 | | 8.56 | | 1.61E-03 | 64.7 | 58.2 | 69.0 | 7.72E-05 | | 67.5 | 78.8 | 60.0 |
| 80 | miR-6816-5p | 9.92 | | 10.16 | | 3.99E-03 | 67.5 | 71.2 | 65.0 | 2.37E-05 | | 72.3 | 84.8 | 64.0 |
| 81 | miR-4741 | 9.74 | | 9.95 | | 4.46E-03 | 64.7 | 65.7 | 64.0 | 3.07E-04 | | 69.9 | 72.7 | 68.0 |
| B) Biliary-tract cancer vs. Healthy control | | | | | | | | | | | | | | |
| Rank | miRNA | | Training cohort | | | | | | | Test cohort | | | | |
|  |  |  | Expression (median in log2) | | | p-value | Accuracy (%) | Sensitivity (%) | Specificity (%) | p-value | Accuracy (%) | | Sensitivity (%) | Specificity (%) |
|  |  |  | Healthy control | | Biliary-tract cancer |  |  |  |  |  |  |  |  |  |
| 1 | miR-125a-3p | | 6.70 | | 4.20 | 1.46E-48 | 97.6 | 95.5 | 99.0 | 9.54E-19 | 98.8 | | 96.9 | 100.0 |
| 2 | miR-6893-5p | | 8.67 | | 7.22 | 2.05E-46 | 95.2 | 92.4 | 97.0 | 6.71E-18 | 95.1 | | 93.8 | 96.0 |
| 3 | miR-204-3p | | 13.29 | | 11.21 | 1.24E-44 | 92.8 | 86.4 | 97.0 | 3.21E-15 | 89.0 | | 93.8 | 86.0 |
| 4 | miR-4294 | | 10.38 | | 9.29 | 3.14E-32 | 90.4 | 83.3 | 95.0 | 5.40E-13 | 84.1 | | 78.1 | 88.0 |
| 5 | miR-6820-5p | | 7.38 | | 6.61 | 2.17E-30 | 90.4 | 89.4 | 91.0 | 8.77E-08 | 84.1 | | 84.4 | 84.0 |
| 6 | miR-150-3p | | 6.92 | | 5.85 | 3.98E-30 | 86.7 | 84.8 | 88.0 | 5.28E-09 | 80.5 | | 90.6 | 74.0 |
| 7 | miR-4476 | | 7.34 | | 5.68 | 7.24E-30 | 86.7 | 83.3 | 89.0 | 8.43E-11 | 87.8 | | 90.6 | 86.0 |
| 8 | miR-6765-3p | | 9.03 | | 7.54 | 2.66E-27 | 87.3 | 80.3 | 92.0 | 9.47E-11 | 85.4 | | 71.9 | 94.0 |
| 9 | miR-6729-5p | | 12.50 | | 12.75 | 1.06E-26 | 85.5 | 78.8 | 90.0 | 1.09E-14 | 87.8 | | 90.6 | 86.0 |
| 10 | miR-7641 | | 7.84 | | 6.02 | 6.32E-26 | 84.9 | 83.3 | 86.0 | 2.18E-11 | 84.1 | | 87.5 | 82.0 |
| 11 | miR-575 | | 6.17 | | 4.63 | 1.05E-24 | 89.2 | 83.3 | 93.0 | 5.01E-11 | 86.6 | | 81.2 | 90.0 |
| 12 | miR-6836-3p | | 8.63 | | 9.21 | 1.10E-22 | 87.3 | 83.3 | 90.0 | 9.33E-07 | 79.3 | | 71.9 | 84.0 |
| 13 | miR-663a | | 10.04 | | 10.57 | 2.93E-22 | 87.3 | 77.3 | 94.0 | 1.07E-10 | 79.3 | | 62.5 | 90.0 |
| 14 | miR-423-5p | | 7.43 | | 6.51 | 1.52E-21 | 83.7 | 80.3 | 86.0 | 9.60E-07 | 76.8 | | 78.1 | 76.0 |
| 15 | miR-1469 | | 10.07 | | 10.50 | 1.74E-21 | 86.1 | 78.8 | 91.0 | 2.05E-08 | 84.1 | | 84.4 | 84.0 |
| 16 | miR-4530 | | 9.50 | | 8.67 | 7.51E-21 | 81.3 | 81.8 | 81.0 | 1.19E-05 | 74.4 | | 81.2 | 70.0 |
| 17 | miR-6075 | | 8.33 | | 9.39 | 1.10E-20 | 83.7 | 69.7 | 93.0 | 3.10E-11 | 84.1 | | 71.9 | 92.0 |
| 18 | miR-4450 | | 6.10 | | 4.60 | 6.36E-19 | 81.3 | 68.2 | 90.0 | 5.71E-06 | 75.6 | | 65.6 | 82.0 |
| 19 | miR-4634 | | 9.74 | | 10.04 | 7.22E-19 | 82.5 | 83.3 | 82.0 | 8.46E-03 | 75.6 | | 78.1 | 74.0 |
| 20 | miR-6877-5p | | 7.26 | | 6.85 | 6.83E-18 | 83.1 | 78.8 | 86.0 | 4.07E-08 | 78.0 | | 71.9 | 82.0 |
| 21 | miR-7109-5p | | 7.51 | | 7.14 | 7.87E-18 | 79.5 | 80.3 | 79.0 | 1.67E-06 | 74.4 | | 75.0 | 74.0 |
| 22 | miR-4454 | | 11.86 | | 11.00 | 1.34E-17 | 82.5 | 77.3 | 86.0 | 3.28E-09 | 85.4 | | 81.2 | 88.0 |
| 23 | miR-6789-5p | | 9.79 | | 10.19 | 5.39E-17 | 79.5 | 78.8 | 80.0 | 1.58E-06 | 80.5 | | 78.1 | 82.0 |
| 24 | miR-7975 | | 10.17 | | 9.46 | 2.94E-15 | 77.1 | 68.2 | 83.0 | 8.19E-07 | 80.5 | | 75.0 | 84.0 |
| 25 | miR-4792 | | 6.38 | | 7.12 | 3.24E-15 | 80.1 | 77.3 | 82.0 | 4.54E-06 | 82.9 | | 84.4 | 82.0 |
| 26 | miR-7977 | | 10.02 | | 9.17 | 4.37E-15 | 78.3 | 68.2 | 85.0 | 6.63E-08 | 79.3 | | 65.6 | 88.0 |
| 27 | miR-6799-5p | | 8.19 | | 7.86 | 6.36E-15 | 76.5 | 80.3 | 74.0 | 1.14E-03 | 76.8 | | 78.1 | 76.0 |
| 28 | miR-16-5p | | 6.47 | | 4.77 | 1.49E-14 | 82.5 | 74.2 | 88.0 | 1.05E-03 | 76.8 | | 62.5 | 86.0 |
| 29 | miR-1238-5p | | 6.32 | | 6.85 | 1.89E-14 | 83.1 | 69.7 | 92.0 | 3.93E-05 | 75.6 | | 56.2 | 88.0 |
| 30 | miR-564 | | 6.19 | | 5.01 | 2.20E-14 | 82.5 | 71.2 | 90.0 | 2.16E-05 | 74.4 | | 56.2 | 86.0 |
| 31 | miR-4734 | | 11.80 | | 12.08 | 4.79E-14 | 76.5 | 80.3 | 74.0 | 7.88E-06 | 72.0 | | 90.6 | 60.0 |
| 32 | miR-8073 | | 6.13 | | 6.73 | 9.28E-14 | 81.3 | 72.7 | 87.0 | 1.26E-06 | 84.1 | | 78.1 | 88.0 |
| 33 | miR-6724-5p | | 9.82 | | 10.15 | 1.89E-13 | 76.5 | 71.2 | 80.0 | 1.50E-07 | 78.0 | | 84.4 | 74.0 |
| 34 | miR-1231 | | 6.37 | | 6.84 | 2.81E-13 | 77.7 | 72.7 | 81.0 | 4.67E-09 | 78.0 | | 84.4 | 74.0 |
| 35 | miR-6880-5p | | 7.38 | | 6.65 | 4.64E-13 | 77.7 | 72.7 | 81.0 | 3.10E-06 | 75.6 | | 75.0 | 76.0 |
| 36 | miR-615-5p | | 6.66 | | 6.19 | 1.25E-12 | 74.7 | 78.8 | 72.0 | 2.06E-08 | 79.3 | | 90.6 | 72.0 |
| 37 | miR-602 | | 6.19 | | 6.54 | 1.40E-12 | 73.5 | 74.2 | 73.0 | 2.18E-04 | 72.0 | | 68.8 | 74.0 |
| 38 | miR-4651 | | 11.00 | | 10.70 | 2.80E-12 | 75.3 | 68.2 | 80.0 | 1.70E-05 | 75.6 | | 65.6 | 82.0 |
| 39 | miR-6090 | | 12.93 | | 13.15 | 6.98E-12 | 74.1 | 71.2 | 76.0 | 1.33E-04 | 74.4 | | 81.2 | 70.0 |
| 40 | miR-4497 | | 12.96 | | 12.37 | 7.22E-12 | 74.1 | 72.7 | 75.0 | 4.17E-06 | 76.8 | | 87.5 | 70.0 |
| 41 | miR-8072 | | 12.22 | | 12.50 | 7.67E-12 | 77.1 | 75.8 | 78.0 | 1.39E-05 | 72.0 | | 71.9 | 72.0 |
| 42 | miR-451a | | 9.03 | | 7.62 | 1.36E-11 | 84.3 | 74.2 | 91.0 | 6.31E-03 | 75.6 | | 53.1 | 90.0 |
| 43 | miR-4723-5p | | 8.96 | | 8.57 | 4.39E-11 | 74.7 | 63.6 | 82.0 | 2.23E-05 | 76.8 | | 71.9 | 80.0 |
| 44 | miR-7114-5p | | 6.93 | | 6.64 | 8.37E-11 | 76.5 | 72.7 | 79.0 | 1.15E-03 | 75.6 | | 68.8 | 80.0 |
| 45 | miR-6726-5p | | 10.04 | | 9.59 | 8.88E-11 | 75.3 | 62.1 | 84.0 | 4.97E-07 | 76.8 | | 75.0 | 78.0 |
| 46 | miR-1227-5p | | 9.44 | | 9.65 | 3.70E-10 | 72.9 | 68.2 | 76.0 | 5.89E-06 | 75.6 | | 78.1 | 74.0 |
| 47 | miR-6850-5p | | 11.26 | | 11.56 | 6.68E-10 | 72.9 | 72.7 | 73.0 | 7.47E-04 | 73.2 | | 87.5 | 64.0 |
| 48 | miR-4732-5p | | 6.29 | | 7.20 | 2.22E-09 | 77.7 | 66.7 | 85.0 | 9.76E-04 | 73.2 | | 56.2 | 84.0 |
| 49 | miR-3622a-5p | | 6.06 | | 5.55 | 3.04E-09 | 80.7 | 59.1 | 95.0 | 7.08E-05 | 74.4 | | 56.2 | 86.0 |
| 50 | miR-8069 | | 12.75 | | 12.96 | 3.36E-09 | 76.5 | 69.7 | 81.0 | 1.20E-06 | 81.7 | | 81.2 | 82.0 |
| 51 | miR-6805-5p | | 11.35 | | 11.53 | 4.69E-09 | 72.3 | 68.2 | 75.0 | 6.15E-05 | 75.6 | | 75.0 | 76.0 |
| 52 | miR-6125 | | 11.86 | | 12.13 | 8.66E-09 | 71.1 | 75.8 | 68.0 | 8.75E-07 | 76.8 | | 87.5 | 70.0 |
| 53 | miR-3188 | | 5.83 | | 6.27 | 9.71E-09 | 75.3 | 72.7 | 77.0 | 2.00E-04 | 74.4 | | 71.9 | 76.0 |
| 54 | miR-6872-3p | | 6.22 | | 5.69 | 1.23E-08 | 74.7 | 71.2 | 77.0 | 8.76E-05 | 76.8 | | 75.0 | 78.0 |
| 55 | miR-4433-3p | | 7.13 | | 7.53 | 4.15E-08 | 73.5 | 74.2 | 73.0 | 1.58E-03 | 75.6 | | 78.1 | 74.0 |
| 56 | miR-6722-3p | | 8.46 | | 8.60 | 2.53E-07 | 72.3 | 68.2 | 75.0 | 5.53E-05 | 73.2 | | 78.1 | 70.0 |
| 57 | miR-6781-5p | | 10.35 | | 10.54 | 3.49E-07 | 69.9 | 74.2 | 67.0 | 4.89E-03 | 69.5 | | 71.9 | 68.0 |
| 58 | miR-1260b | | 8.71 | | 8.32 | 4.12E-07 | 68.7 | 60.6 | 74.0 | 1.59E-03 | 75.6 | | 65.6 | 82.0 |
| 59 | miR-1260a | | 7.05 | | 6.68 | 6.94E-07 | 67.5 | 62.1 | 71.0 | 3.95E-03 | 72.0 | | 65.6 | 76.0 |
| 60 | miR-4486 | | 6.97 | | 7.30 | 1.59E-06 | 66.9 | 63.6 | 69.0 | 8.49E-06 | 75.6 | | 78.1 | 74.0 |
| 61 | miR-4707-5p | | 7.18 | | 7.43 | 1.61E-06 | 68.7 | 63.6 | 72.0 | 4.00E-03 | 76.8 | | 71.9 | 80.0 |
| 62 | miR-3185 | | 6.70 | | 7.16 | 2.01E-06 | 68.7 | 62.1 | 73.0 | 1.29E-05 | 72.0 | | 78.1 | 68.0 |
| 63 | miR-3135b | | 7.93 | | 7.50 | 8.55E-06 | 70.5 | 77.3 | 66.0 | 1.16E-04 | 81.7 | | 87.5 | 78.0 |
| 64 | miR-4534 | | 6.82 | | 6.52 | 2.44E-04 | 71.7 | 63.6 | 77.0 | 4.06E-03 | 68.3 | | 71.9 | 66.0 |
| 65 | miR-1915-3p | | 10.93 | | 11.12 | 2.27E-03 | 64.5 | 66.7 | 63.0 | 6.83E-05 | 76.8 | | 81.2 | 74.0 |
| 66 | miR-4687-3p | | 9.59 | | 9.46 | 2.90E-03 | 65.7 | 60.6 | 69.0 | 4.92E-03 | 73.2 | | 65.6 | 78.0 |
