# Supplementary material for: MicroRNA Markers for the Diagnosis of Pancreatic and Biliary-Tract Cancers
Source: PLoS One. 2015 Feb 23;10(2):e0118220. doi: 10.1371/journal.pone.0118220 (PMC4338196; doi:10.1371/journal.pone.0118220)
Supplement: S2 Table — (DOCX) [file pone.0118220.s002.docx]

SUPPORTING INFORMATION

S2 Table. Best discriminate functions for each possible number of miRNAs used in the test cohort.

| Number of miRNAs used | Best discriminant functions |
| --- | --- |
| 1 | 0 = 2.692 x miR-6836-3p - 24.022 |
| 2 | 0 = 1.550 x miR-6075 + 1.236 x miR-6836-3p - 24.911 |
| 3 | 0 = 1.383 x miR-6075 + 0.980 x miR-6836-3p - 0.329 x miR-4476 - 19.059 |
| 4 | 0 = 1.202 x miR-6075 - 0.927 x miR-6799-5p - 0.220 x miR-125a-3p + 0.711 x miR-6836-3p - 8.549 |
| 5 | 0 = 1.132 x miR-6075 - 0.143 x miR-4294 - 0.831 x miR-6799-5p - 0.205 x miR-125a-3p + 0.701 x miR-6836-3p - 7.289 |
|  | 0 = 1.175 x miR-6075 - 0 .818 x miR-6799-5p - 0.130 x miR-125a-3p - 0.386 x miR-4530 + 0.681 x miR-6836-3p - 5.843 |
| 6 | 0 = 1.147 x miR-6075 - 0.060 x miR-4294 - 0.782 x miR-6799-5p - 0.127 x miR-125a-3p - 0.373 x miR-4530 + 0.678 x miR-6836-3p - 5.406 |
|  | 0 = 1.041 x miR-6075 - 0.257 x miR-6880-5p - 0.779 x miR-6799-5p - 0.137 x miR-125a-3p + 0.586 x miR-6836-3p - 0.175 x miR-4476 - 4.649 |
| 7 | 0 = 1.052 x miR-6075 - 0.218 x miR-6880-5p - 0.749 x miR-6799-5p - 0.102 x miR-125a-3p - 0.191 x miR-4530 + 0.588 x miR-6836-3p - 0.156 x miR-4476 - 3.844 |
| 8 | 0 = 1.034 x miR-6075 - 0.040 x miR-4294 - 0.216 x miR-6880-5p - 0.725 x miR-6799-5p - 0.100 x miR-125a-3p - 0.183 x miR-4530 + 0.586 x miR-6836-3p - 0.156 x miR-4476 - 3.560 |
| 9 | 0 = 1.142 x miR-6075 + 0.222 x miR-4294 - 0.101 x miR-6880-5p - 0.473 x miR-6799-5p - 0.057 x miR-125a-3p - 0.433 x miR-4530 + 0.322 x miR-6836-3p - 2.189 x miR-7114-5p - 0.259 x miR-4476 + 9.896 |
|  | 0 = 1.077 x miR-6075 + 0.153 x miR-4294 - 0.191 x miR-6880-5p - 0.454 x miR-6799-5p - 0.128 x miR-125a-3p + 0.261 x miR-6836-3p + 0.325 x miR-4634 - 2.074 x miR-7114-5p - 0.300 x miR-4476 + 4.836 |
| 10 | 0 = 1.138 x miR-6075 + 0.231 x miR-4294 - 0.106 x miR-6880-5p - 0.434 x miR-6799-5p - 0.057 x miR-125a-3p - 0.425 x miR-4530 + 0.272 x miR-6836-3p + 0.266 x miR-4634 - 2.150 x miR-7114-5p - 0.262 x miR-4476 + 7.060 |
